# Systolic time ratio measured by impedance cardiography accurately screens left ventricular diastolic dysfunction in patients with arterial hypertension

**DOI:** 10.1186/s40885-017-0084-y

**Published:** 2017-12-27

**Authors:** Rodrigo Nazário Leão, Pedro Marques Silva, Luísa Branco, Helena Fonseca, Bruno Bento, Marta Alves, Daniel Virella, Roberto Palma Reis

**Affiliations:** 1 Unidade Funcional Medicina 1.2, Hospital de São José, Centro Hospitalar Lisboa Central-EPE, Rua José António Serrano, 1150-199 Lisboa, Portugal; 2 NOVA Medical School, Universidade NOVA de Lisboa, Lisboa, Portugal; 3 Núcleo de Investigação Arterial, Unidade Funcional Medicina 4, Hospital Santa Marta, Centro Hospitalar Lisboa Central-EPE, Lisboa, Portugal; 4 Laboratório de Ecocardiografia, Serviço de Cardiologia, Hospital de Santa Marta, Centro Hospitalar de Lisboa Central-EPE, Lisboa, Portugal; 5 Unidade de Cardiologia, Hospital Pulido Valente, Centro Hospitalar Lisboa Norte-EPE, Lisboa, Portugal; 6 Gabinete de Análise Epidemiológica e Estatística, Centro de Investigação, Centro Hospitalar Lisboa Central-EPE, Lisboa, Portugal

**Keywords:** Arterial hypertension, Diastolic dysfunction, Impedance Cardiography, Systolic time ratio, Screening

## Abstract

**Background:**

The use of impedance cardiography (ICG) may play a role in the assessment of cardiac effects of hypertension (HT), especially its hemodynamic features. Hypertensive heart disease involves structural changes and alterations in left ventricular geometry that end up causing systolic and/or diastolic dysfunction. The *IMPEDDANS* study aims to assess the usefulness of ICG for the screening of left ventricular diastolic dysfunction (LVDD) in patients with HT.

**Methods:**

Patients with HT were assessed by echocardiography and ICG. Receiver-operating characteristic curve and the area under the curve were used to assess the discriminative ability of the parameters obtained by ICG to identify LVDD, as diagnosed by echocardiography.

**Results:**

ICG derived pre-ejection period (PEP), left ventricle ejection time (LVET), systolic time ratio (STR) and D wave were associated (*p* < 0.001) with LVDD diagnosis, with good discriminative ability: PEP (AUC 0.81; 95% CI 0.74–0.89), LVET (AUC 0.82; 95% CI 0.75–0.88), STR (AUC 0.97; 95% CI 0.94–1.00) and presence of D wave (AUC = 0.87; 95% CI 0.82–0.93). STR ≥ 0.30 outperformed the other parameters (sensitivity of 98.0%, specificity of 90.2%, positive predictive value of 95.2%, and negative predictive value of 96.1%).

**Conclusion:**

The ICG derived value of STR allows the accurate screening of LVDD in patients with HT. It might as well be used for follow up assessment.

**Trial registration:**

The study protocol was retrospectively registered as IMPEDDANS on ClinicalTrials.gov (ID: NCT03209141) on July 6, 2017.

## Background

Arterial hypertension (HT) causes high morbidity and mortality [1]. The progression of hypertensive heart disease involves myocardial fibrosis and changes in left ventricular geometry that precedes functional changes [2]. Diastolic dysfunction is part of this continuum, and despite the growing recognition of its importance, it is generally undervalued because of the difficulty in its diagnosis and the absence of effective therapies [3]. Currently, the asymptomatic cardiac damage repercussion of HT is mainly evaluated by electrocardiography and by echocardiography [4]. Cardiac magnetic resonance imaging (MRI) is an alternative diagnostic examination but should only be considered for assessment of left ventricle (LV) size and mass when echocardiography is not feasible (or, in less frequent situations, when imaging of delayed enhancement could have therapeutic implications). Impedance cardiography (ICG) is a non-invasive, non-operator dependent, low-cost complementary diagnostic tool that easily allows characterizing a hypertensive patient’s hemodynamic phenotypic profile and optimizing the antihypertensive therapy [5, 6]. It analyses and registers hemodynamic changes through the measurement of electrical resistance changes in the thorax and translating them graphically as impedance and electrocardiogram waveforms [5, 7, 8]. This technique has evolved in recent years, making it an attractive and economical alternative approach. However, it is recognized that well-designed clinical trials are lacking in well-defined populations, in order to justify its widespread use in clinical practice [9–12]. Some other more specific procedures are reserved only for diagnosis of myocardial ischaemia in hypertensive patients with LV hypertrophy [4]. Therefore it is important to have alternative tests for the initial assessment of diastolic function in hypertensive patients.

In diastolic dysfunction, there is a relative shift in LV filling to the final part of the diastole with a greater dependence on atrial contraction. These changes can be detected by Doppler echocardiography and by ICG [2].

**Fig. 1:**
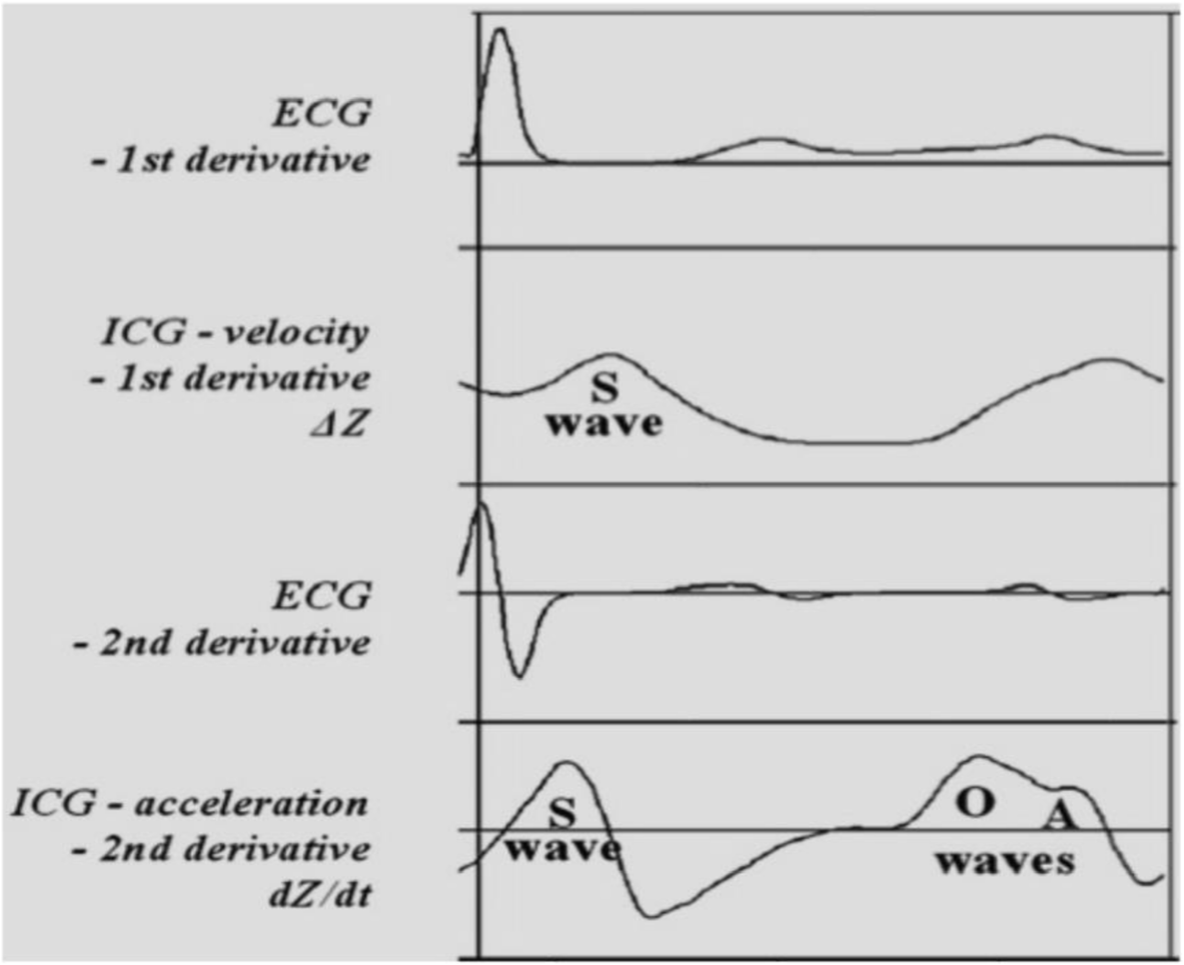
ICG waves in diastolic dysfunction (adapted from Bour et al. [5])

The phase of rapid passive ventricular filling at the onset of diastole produces the E wave and the atrial contraction produces the A wave. The E and the A waves on the Doppler sonography correspond to the O and A waves in ICG, respectively [13, 14]. Normally, the E wave in Doppler is higher than the A wave, whereas in diastolic dysfunction, the E wave is prolonged and becomes smaller than the A wave. ICG can detect diastolic dysfunction by revealing a delayed O wave (called a D wave) when contractility is normal, [14] whereas diastolic dysfunction produces a significant increase in the amplitude and duration of A wave (Fig. 1) [5]. A D wave with an amplitude above the dZ/dt baseline greater than one third of the amplitude of the corresponding E wave is considered abnormal [15].

The pre-ejection period (PEP), corresponding to the interval between the depolarization onset (Q-wave) and aortic valve opening that includes the excitation-contraction coupling and isovolumetric contraction of the LV, decreases with increasing LV pressure at the end of diastole, the main physiological consequence of diastolic dysfunction [16–18]. On the other hand, the left ventricular ejection time (LVET), measured as the interval between the opening of the aortic valve and its closure, increases in patients with diastolic dysfunction. It has been reported that a marked increase in LVET occurs in patients with isolated diastolic dysfunction and that this increase correlates with the severity of diastolic dysfunction [19]. Thus, in diastolic dysfunction, the systolic time ratio (STR) is expected to decrease (STR = PEP / LVET).

The thoracic fluid content (TFC) reflects the total volume of fluid (intravascular and extravascular) within the thoracic cavity, being inversely related to patient transthoracic electrical bioimpedance. It is therefore a reliable index of fluid overload in ambulatory patients. This parameter increases with the evolution of diastolic dysfunction being a sign of severity and has recently been proposed for monitoring diastolic heart failure patients [20, 21].

The purpose of IMPEDDANS study is to assess the usefulness of ICG in the screening of LV diastolic dysfunction (LVDD) in patients with HT.

## Methods

This is a validation study of a diagnostic method (ICG) used for a new purpose (diagnosis of LVDD) comparing it with the clinical standard (echocardiography), the method currently used for this effect in usual clinical practice.

Patients of either gender, aged 18–75 years, diagnosed with grade 2 or 3 HT (systolic blood pressure [BP] ≥ 160 mmHg and/or diastolic BP ≥ 100 mmHg) and/or with resistant hypertension (according the definition of ESH/ESC guidelines), [4] followed up in ambulatory clinic of an Internal Medicine Department of a tertiary referral hospital were eligible for recruitment.

Patients were excluded in the presence of: pregnancy, height less than 120 cm or more than 230 cm, weight less than 30 kg or greater than 155 kg, heart failure II-IV NYHA, [22] heart rate (HR) less than 50 bpm or greater than 110 bpm, atrial fibrillation or flutter, more than three premature ventricular contractions per hour, complete left bundle branch or atrioventricular block, pacemaker, severe valvulopathy, constrictive pericarditis, hypertrophic and restrictive cardiomyopathy, ischaemic heart disease and/or segmental kinetics anomalies assessed by echocardiography, left ejection fraction <50%, or poor echocardiographic window.

Recruitment began in January 2015 and was finished in July 2017.

Sample size was estimated to test a hypothesized positive predictive value of 70 ± 5% of the ICG parameters with 95% confidence. Since the prevalence of LVDD in patients with HT is estimated, in most studies, [2, 23, 24] to be approximately 50%, to reach an effective sample of 77 patients with LVDD, the sample to be recruited was doubled to 154 hypertensive patients.

Considering that it would not be ethically acceptable to perform right heart catheterization of patients with grade 2 or 3 HT, we chose to design a concordance study between ICG and echocardiography, which is the clinical standard for the diagnosis of LVDD and characterization of hypertensive cardiomyopathy.

Participants were invited to be systematically assessed by ICG and echocardiography, within a maximum interval of 8 days. To ensure that both tests were performed under similar conditions, evaluations matching variations greater than 10% in BP or variations more than 5% in HR were not considered. These patients were asked to repeat at least one of the exams. If the variations persisted, they were excluded.

Demographic and clinical baseline data were collected from the patients’ medical records. Outpatient HT clinic protocol requires clinical evaluation, blood chemistry tests, electrocardiogram (EKG) and, eventually, 24 h ambulatory blood pressure monitoring (AMBP). Data regarding the comorbidities and medication were collected. Body mass index was calculated as weight (kg) divided by height (m) squared.

ICG was carried out by an experienced cardiopneumology technician with Niccomo – Non-Invasive Continuous Cardiac Output Monitor (Medis, GmbH, Ilmenau, Germany) according the approved department protocol. According to this, patients had to present with 6 h of fasting but took their usual antihypertensive drugs. It was carried out in supine position during 20 min (continuous recording) and 70° orthostatism with the help of the tilting table (10 min in continuous recording). The test was interrupted if there was syncope or pre-syncope; dizziness, nausea and malaise associated with poorly tolerated hypotension and/or bradycardia; pain/precordial discomfort; EKG ST segment changes; systolic BP > 210 mmHg. Table 1 presents the evaluated parameters.

**Table 1:** Parameters assessed in impedance cardiography

Parameter	Definition	Formula
Heart rate	Number of heart beats per minute (bpm)	RR interval measurement on the ECG and extrapolation for bpm
Mean blood pressure (MAP)	Average pressure exerted by the blood on the arterial walls	Automatic (oscillometric method) = MAP is measured directly and SBP and DBP are derived
Cardiac output (CO)	Amount of blood ejected from the left ventricle / minute	CO = Stroke volume x Heart rate
Cardiac index (CI)	Standard CO for the body surface area (BSA)	CI = CO / BSA
Stroke volume (SV)	Amount of blood ejected from the left ventricle / heart beat	SV = VEPT x LVET x VI (Z MARC Algorithm)
Stroke volume index (SVI)	Standard SV for the BSA	SVI = SV / BSA
Vascular systemic resistance (VSR)	Resistance to circulating blood in the arterial system	VSR = 80 x ((MAP-CVP)/CO)
Vascular systemic resistance index (VSRI)	Standard resistance to circulating blood in the arterial system for the BSA	VSRI = 80 x ((MAP-CVP)/CI)
Acceleration index (AI)	Initial acceleration of blood in the aorta that occurs within the first 10–20 milliseconds after opening of the aortic valve	AI = (d^2^Z/dt^2^_Max_)/TFI
Velocity index (VI)	Aorta blood velocity peak	VI = (dZ/dt_Max_)/TFI
Thoracic fluid (TF)	Electrical conductivity of the thoracic cavity (determined by intravascular, interalveolar and interstitial fluids)	TF = 1/TFI
Left heart work (LHW)	Indicator of the amount of work the left ventricle exerts to pump blood every minute	LHW = (MAP – PAOP) x CO
Left heart work index (LHWI)	Standard LHW for the BSA	LHW = (MAP – PAOP) x CI
Systolic time ratio (STR)	Ratio of electrical and mechanical systole	STR = PEP / LVET
Pre-ejection period (PEP)	Time interval from the beginning of the electrical stimulation of the ventricles to the beginning of the opening of the aortic valve (electric systole)	Time interval between the start of wave Q on the ECG and point B on wave dZ / dt (opening of the aortic valve)
Left ventricular ejection time (LVET)	Time interval from opening to closing of the aortic valve (mechanical systole)	Time interval from point B to point X on wave dZ / dt

*CVP* Central venous pressure (pressure in the thoracic and right atrial vein - 6 mmHg is considered by default), *dZ / dt*_*Max*_ Maximum of the first derivative of ΔZ, *d* ^*2*^ *Z / dt* ^*2*^_*Max*_ Maximum of the second derivative of ΔZ, *PAOP* Pulmonary artery occlusion pressure (considered by default as 10 mmHg), *TFI* Thoracic fluid index (baseline thoracic impedance, Z_0_), *VEPT* Volume of electrically participating tissue (conductive volume for chest size affected by weight, height and sex)

**Fig. 2:**
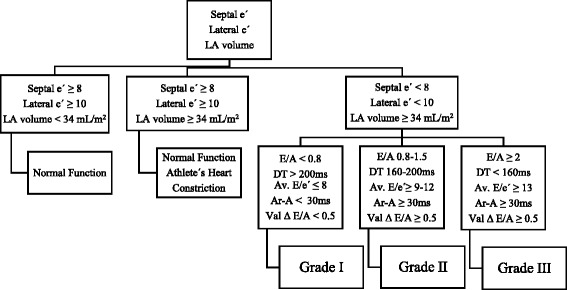
Practical approach to grade diastolic dysfunction (adapted from Nagueh et al. [25])

Transthoracic echocardiography was performed in Vivid E9 and S5 devices (GE Healthcare, Chicago, Illinois, USA), always by the same three experienced cardiologists. To ensure uniformity of evaluation and correct evaluation, all exams were reviewed by one of the other cardiologists. In order to diagnose and classify diastolic dysfunction, the left atrium volume index, the velocities E, A, septal e’, lateral e’, deceleration time (DT), isovolumetric relaxation time (IRVT), atrial reverse velocity (Ar) and E/A ratio variation with Valsalva manoeuvre were recorded (whenever the patient cooperated and if the images obtained had the necessary quality for analysis) as recommended by 2009 guidelines. We considered that patients had LVDD when present septal e’ < 8, lateral e’ < 10 and left atrial volume equal or greater than 34 mL/m^2^ (Fig. 2) [25].

The estimated efficacy and effectivity measures are the positive predictive value (PPV), negative predictive value (NPV), sensitivity and specificity of the presence of the D wave and the values of PEP, LVET, STR, and TFC obtained by ICG to detect LVDD.

Categorical variables were described with frequencies and percentages. After evaluating the normal distributions of the continuous variables by the Kolmogorov-Smirnov test and histograms analysis, the variables that presented normal distribution were described with mean and standard deviation, and the variables without normal distribution were described with median and interquartile range (25th percentile, P_25_ - 75th percentile, P_75_) or amplitude (minimum - maximum).

To compare the ICG parameters between patients with and without LVDD, Mann-Whitney test was used. Cut-off points were determined for ICG parameters (PEP, LVET and STR) as predictors of occurrence of LVDD. Using the receiver-operating characteristic (ROC) curve cut-off points were determined regarding the diagnosis of LVDD capacity for screening purposes, thus high sensitivity was considered (>90%). The area under the curve (AUC) was estimated and 95% confidence intervals calculated using the binomial exact confidence interval method [26]. Dichotomized ICG parameters by the obtained cut-off points, were used to estimate the test performance measures (sensitivity, specificity, positive and negative predictive values as well as false positive and negative rates). The level of significance α = 0.05 was considered. Data were analysed with SPSS 22.0 (SPSS for Windows, Rel. 22.0.1. 2013. SPSS Inc., Chicago, Il, EUA) and MedCalc Statistical Software version 16.4.3 (MedCalc Software bvba, Ostend, Belgium; https://www.medcalc.org; 2016).

The study protocol was approved by the hospital’s Ethics Committee (approval number 166/2014). Informed consent was obtained from each patient.

The study protocol is registered as IMPEDDANS on ClinicalTrials.gov (ID: NCT03209141).

## Results

IMPEDDANS study recruited 167 patients. Ten patients were excluded: three had syncope during ICG, three gave up and did not perform the tests, three had a significant dysrhythmia and one was diagnosed with atrial septal defect. From the 157 hypertensive patients included, 102 (65%) had LVDD (diagnosed by echocardiogram). Table 2 shows the main demographic, clinical and hemodynamic characteristics of the patients.

**Table 2:** Main characteristics of the effective sample (*n* = 157)

	All patients (*n* = 157)	Without LVDD (*n* = 55)	With LVDD (*n* = 102)
Males, n (%)	88 (56.1)	28 (50.9)	60 (58.8)
Age, mean (SD)	63 (10)	58 (11)	65 (9)
Caucasian, n (%)	142 (90.4)	48 (87.3)	94 (92.1)
HT duration (months), mean (SD)	120 (104)	47 (53)	160 (104)
Resistant HT, n (%)	117 (74.5)	41 (74.5)	76 (74.5)
Obesity, n (%)	82 (52.2)	31 (56.4)	51 (50.0)
Diabetes Mellitus, n (%)	81 (51.6)	28 (52.8)	53 (51.9)
Dyslipidaemia, n (%)	133 (84.7)	47 (85.5)	86 (84.3)
CKD, n (%)	49 (31.2)	12 (21.8)	37 (36.3)
Stroke, n (%)	36 (22.9)	13 (23.6)	23 (22.5)
COPD, n (%)	20 (12.7)	5 (9.1)	15 (14.7)
OSA, n (%)	31 (19.7)	8 (14.5)	23 (22.5)
Smoker, n (%)	34 (21.7)	6 (10.9)	28 (27.5)
Alcoholic, n (%)	16 (10.8)	6 (10.9)	10 (98.0)
N° of AHT drugs, median (min-max)	4 (1–7)	4 (1–7)	4 (1–6)
SBP (mmHg), median (P_25_-P_75_)	131 (122–142)	124 (118–135)	133 (126–144)
DBP (mmHg), median (P_25_-P_75_)	78 (72–84)	78 (73–84)	77 (72–84)
MBP (mmHg), median (P_25_-P_75_)	91 (85–99)	91 (84–96)	92 (85–101)
HR (bpm), median (P_25_-P_75_)	63 (57–70)	66 (60–74)	62 (56–69)
SVI (ml/m^2^), median (P_25_-P_75_)	43 (35–51)	37 (31–45)	46 (39–54)
CI (l/min/m^2^), median (P_25_-P_75_)	2.7 (2.4–3.1)	2.6 (2.3–2.8)	2.8 (2.5–2.4)
LCWI (kg.m/m^2^), median (P_25_-P_75_)	3.3 (2.7–3.8)	3.1 (2.6–3.5)	3.4 (2.9–4.4)
SVRI (dyne.s.cm^5^/m^2^), median (P_25_-P_75_)	2599 (2212–3083)	2797 (2477–3185)	2472 (2142–2922)
VI (1/1000/s), median (P_25_-P_75_)	39 (31–48)	36 (29–44)	40 (31–49)

*AHT* antihypertensive, *CI* cardiac index, *CKD* chronic kidney disease, *COPD* chronic obstructive pulmonary disease, *DBP* diastolic blood pressure, *HR* heart rate, *LCWI* left cardiac work index, *LVDD* left ventricle diastolic dysfunction, *MBP* mean blood pressure, *OSA* obstructive sleep apnoea, *SBP* systolic blood pressure, *SVI* stroke volume index, *SVRI* systemic vascular resistance index, *VI* Aorta velocity index

**Fig. 3:**
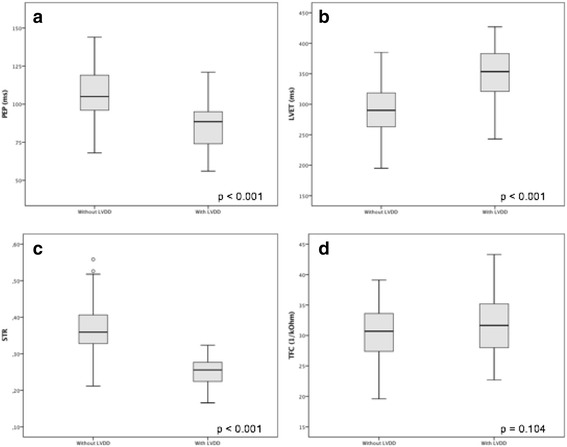
Distribution of the values of (**a**) pre-ejection period (PEP), **b** left ventricle ejection time (LVET), **c** systolic time ratio (STR) and **d** thoracic fluid content (TFC), as assessed by ICG, according with the diagnosis of LVDD (left ventricular diastolic dysfunction) by echocardiography. Graphics represent the interquartile range (P_25_-P_75_), median, limits and outliers. Comparison by Mann-Whitney test

The patients with LVDD diagnosis had significantly lower PEP and STR and higher LVET and TFC than the patients without LVDD (Table 3 and Fig. 3). The D wave was more frequently identified among patients with LVDD (Table 3) and its presence was also associated with LVDD diagnosis.

**Table 3:** Distribution of the values of measurements obtained by ICG considered to be used to screen LVDD (*n* = 157). Mann-Whitney test

	All patients (*n* = 157)	Without LVDD (*n* = 55)	With LVDD (*n* = 102)	*p*
PEP (ms), median (P_25_-P_75_)	93 (80–105)	105 (96–120)	88 (74–95)	< 0.001
LVET (ms), median (P_25_-P_75_)	332 (291–370)	290 (262–320)	353 (320–383)	< 0.001
STR, median (P_25_-P_75_)	0.28 (0.21–0.33)	0.36 (0.32–0.41)	0.26 (0.22–0.28)	< 0.001
TFC (1/kΩ), median (P_25_-P_75_)	31.3 (27.6–34.7)	30.7 (27.4–34.0)	31.6 (27.9–35.2)	0.104
D wave, n (%)	83 (52.9)	2 (1.9)	81 (79.4)	< 0.001

*ICG* impedance cardiography, *LVDD* left ventricular diastolic dysfunction, *LVET* left ventricle ejection time, *PEP* pre-ejection period, *STR* systolic time ratio, *TFC* thoracic fluid content

**Fig. 4:**
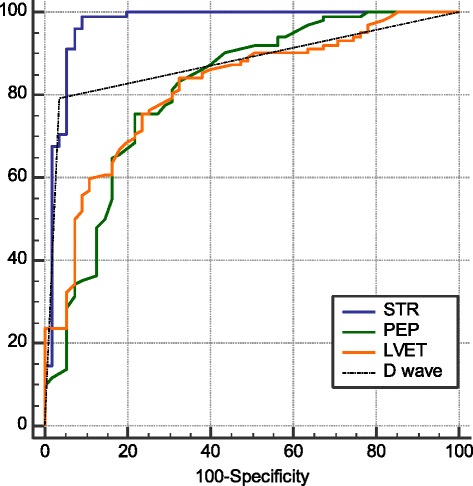
Discriminative ability of PEP, LVET, STR and D wave to identify LVDD in hypertensive patients, as determined by the AUC and ROC curves. STR AUC = 0.97; PEP AUC = 0.81; LVET AUC = 0.82; D Wave AUC = 0.88. AUC, area under the curve; LVDD, left ventricular diastolic dysfunction; LVET, left ventricle ejection time; PEP, pre-ejection period; STR, systolic time ratio

The diagnostic performance was estimated for those ICG parameters significantly associated with the diagnosis of LVDD. The AUC estimated for PEP was 0.81 (95% CI 0.74–0.87; *p* < 0.001), for LVET the AUC was 0.82 (95% CI 0.75–0.87; *p* < 0.001), the AUC was 0.97 (95% CI 0.93–0.99; *p* < 0.001) for STR and for the presence D wave the AUC was 0.88 (95% CI 0.82–0.93; *p* < 0.001) (Fig. 4). From the analysis of the ROC curves, were defined the following cut-off points for LVDD screening: PEP ≤ 104 ms, LVET ≥ 320 ms; STR ≤ 0.31. Table 4 presents the results of the LVDD diagnostic evaluation with the ICG variables PEP, LVET, STR and D wave.

**Table 4:** Diagnostic performance of ICG-derived indexes for identification of LVDD, for the identified cut-off points (*n* = 157)

Index and cut-off	TP	FP	TN	FN	Sensitivity % (95% CI)	Specificity % (95% CI)	PPV % (95% CI)	NPV % (95% CI)
PEP ≤ 104 ms	92	24	31	10	90.2 (82.7–95.2)	56.4 (42.3–69.7)	79.3 (73.8–83.9)	75.6 (62.2–85.4)
LVET ≥ 290 ms	92	28	27	10	90.2 (82.7–95.2)	49.1 (35.4–62.9)	76.6 (71.6–81.1)	72.9 (58.6–83.8)
STR ≤ 0.31	101	5	50	1	99.0 (94.7–99.9)	90.9 (80.0–96.9)	95.3 (89.8–97.9)	98.0 (87.7–99.7)
D wave presence	81	2	53	21	79.4 (70.3–86.8)	96.4 (87.5–99.6)	97.6 (91.2–99.4)	71.6 (63.2–78.8)

*FN* false negative diagnostics, *FP* false positive diagnostics, *NPV* negative predictive value, *LVDD* left ventricular diastolic dysfunction, *LVET* left ventricle ejection time, *PEP* pre-ejection period, *PPV* positive predictive value, *STR* systolic time ratio, *TN* true negative diagnostics, *TP* true positive diagnostics

The misclassification of LVDD using STR affected 6 cases, one false negative (FN) and five false positive (FP). The only FN case had STR = 0.32, which is only slightly above the cut-off value 0.31. No demographic, clinical and hemodynamic characteristics were found that differentiated the misclassified cases from the rest of the sample.

## Discussion

In the IMPEDDANS study, the STR, with sensitivity of 99.0% and specificity of 90.9%, outperformed the other analysed variables as screening test for LVDD. This index only misclassified 5 FP cases, which determine a 95.3% PPV, and 1 FN case, which determines a 98.0% NPV.

In the IMPEDDANS study, PEP ≤ 104 ms was identified as a good screening test for LVDD with sensitivity of 90.2% and PPV of 79.3%. PEP extends from the onset of ventricular depolarization to the opening of the aortic valve. It is composed of two sub-intervals, known as the period of the electromechanical coupling and the period of elevation of pressure (or isovolumetric systole). The electromechanical coupling period undergoes few changes in acute interventions, but changes in the final diastolic pressure, aortic diastolic pressure and in the mean velocity of elevation of LV pressure (usually predominantly) in this period are the factors that determine the period of pressure rise and, consequently, PEP. Elevation of LV end-diastolic pressure (LVEDP), reduction of aortic diastolic pressure or gain in the rate of pressure increase in the pre-ejection period, lead to a time reduction. In mild LVDD, we expect a rise in LVEDP and a shortening of PEP, as we confirmed in this sample [16–18]. However, we must point out that LVEDP, aortic diastolic pressure or alterations in the rate of pressure increase can act simultaneously and we can have an unexpected PEP time. For example, in the established heart failure, the final diastolic pressure is increased, but the rate of ventricular pressure elevation in the pre-ejective period is so slow that it predominates and prolongs PEP. In this sample, it was not the case, because we excluded the patients with heart failure [16–18].

Increased LVET (**≥** 320 ms) was also a good LVDD screening tool with sensitivity 90.2%, and PPV 76.6%. Considering that active relaxation can be considered in the strictest sense as an early diastolic event, the time of onset of this process depends, at least in part, on systolic events such as the duration of contraction. In LVDD, increases in load during the contraction phase induce compensatory increases in duration of systole (LVET) and delayed onset of relaxation, which explain these results [27, 28].

The diastolic properties of the LV are largely determined by the size or volume of the LV, the thickness, physical properties of the ventricular wall and the relaxation process of the myocardium. Thus, a combination of increased myocardial mass and changes in the extracellular collagen network may cause or contribute to an increase in the passive elastic rigidity of the ventricle with a diastolic pressure-volume relation represented by a steep curve. Disorders of the active myocardial relaxation process, acting alone or in conjugation with abnormal passive ventricular properties, may also cause increased ventricular rigidity. As a result, there is a reduction in the compliance and distensibility of the ventricle, the filling dynamics are changed and the final diastolic pressure increases. Under these circumstances, as the disease progresses, an increase in central blood volume may produce a substantial increase in LV diastolic pressure and, consequently, cause some degree of pulmonary congestion, which can be evidenced by an increase in TFC [13, 29, 30]. In the IMPEDDANS study, however, we did not verify any relation between LVDD and TFC. We hypothesize that this is related with the important proportion of resistant hypertensive patients which are polymedicated (*n* = 117, 74.5% of the total sample; 48.4% of the patients with LVDD, *n* = 76), most of them taking diuretics (*n* = 82, 80.4%). On the other hand, pulmonary congestion is more common in an advanced phase of hypertensive disease, when patients start to evidence heart failure symptoms, which were not included (only patients with mild asymptomatic LVDD were included).

During early diastole, the occurrence of a prominent wave may indicate diastolic dysfunction. As such, the presence of a pronounced diastolic O wave (D wave), in either upright or supine position, can be assumed as an evidence of diastolic dysfunction [2]. Thus, it was verified that the presence of a D wave was statistically related with LVDD and that it had good performance screening LVDD, with 79.4% of sensitivity and 97.6% PPV.

Our observational study has some weak points: the two tests were performed within a time lapse of 8 days. In order to minimize this weakness, evaluations matching variations >10% in BP or variations >5% in HR were not considered. Although sample was recruited by convenience of researchers all patients had strict inclusion and exclusion criteria, were submitted to the same evaluation protocols and patients were recruited non-consecutively. Significant strengths are the exclusion of the elderly (due to the influence of age on the physiopathology of diastolic dysfunction), and the fact that examinations have been performed in a clinical environment controlled by experienced professionals under well-defined protocols.

**Fig. 5:**
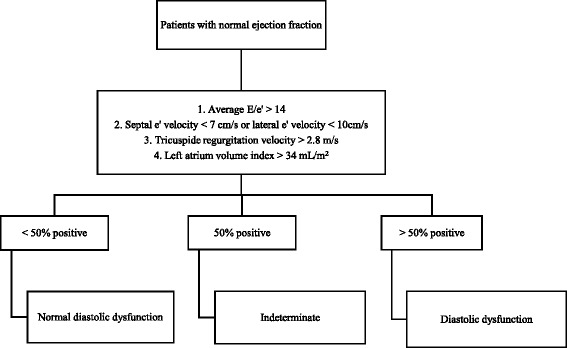
Practical approach to diagnose diastolic dysfunction (adapted from Nagueh et al. [31])

Recommendations for the diagnosis of diastolic dysfunction were updated in 2016 (Fig. 5 presents the diagnostic approach proposed) [31]. However, there is no real consensus in the scientific community regarding its applicability due to the fact that they have not yet been duly validated and just during this year was presented the first trial studying their applicability being difficult to conclude about their performance [32, 33]. We can, however, admit that the new recommendations create situations in which diastolic dysfunction may be underdiagnosed. In fact, the inclusion of the tricuspid regurgitation peak velocity as a criterion for the diagnosis and classification of diastolic dysfunction may lead to some interpretation problems because high values may be due to pulmonary hypertension and its increase is usually associated with elevation of pulmonary artery pressure due to increased filling pressures of the left ventricle, phenomena present in advanced stages of diastolic dysfunction [34–37]. Thus, we can only diagnose diastolic dysfunction in patients with established heart failure and patients with diastolic dysfunction at an early stage may go unnoticed, which goes against the spirit of this study, whose aim is to diagnose patients at an early stage, asymptomatic, without heart failure. In addition, the adoption of new recommendations would bring the limitation of not allow to compare our results with previous studies and perform other substudies with the acquired data. After analyzing these points and considering that these recommendations were published in the middle of the study period, we chose to not integrate them into our protocol and to keep the previous recommendations, already studied and validated.

Despite all the potential demonstrated by the ICG, we must emphasize the importance of performing routine echocardiography in hypertensive patients, indispensable to provide the recommended basic echocardiographic assessments [4]. In hypertensive patients ICG can be a valid, cost effective alternative for LVDD screening, it might also be used to monitor follow-up and define patients who would benefit from early transthoracic echocardiography.

## Conclusions

ICG can accurately screen LVDD in hypertensive patients, using its derived parameters STR, PEP, LVET and D wave. STR alone showed the highest discriminative ability. Therefore, we recommend its use as a LVDD screening tool.

Future ICG studies applying the diagnostic test for LVDD will probably increase our understanding of the physiopathology/hemodynamics of HT and demonstrate that earlier detection and treatment of these hemodynamic features will favour patients’ evolution and, perhaps, reduce morbidity and mortality.

## Abbreviations

AHT: Antihypertensive
AMBP: 24 hours ambulatory blood pressure monitoring
Ar: Atrial reverse velocity
AUC: Area under the curve
BO: Blood pressure
BSA: Body surface area
CI: Confidence interval
CIn: Cardiac index
CKD: Chronic kidney disease
CO: Cardiac output
COPD: Chronic obstructive pulmonary disease
CVP: Central venous pressure
DBP: Diastolic blood pressure
DT: Deceleration time
EKG: Electrocardiogram
FN: False negatives
FP: False positives
HR: Heart rate
HT: Hypertension
ICG: Impedance cardiography
IRVT: Isovolumic relaxation time
LCW: Left cardiac work
LCWI: Left cardiac work index
LV: Left ventricle
LVDD: Left ventricle diastolic dysfunction
LVEDP: Left ventricle end diastolic pressure
LVET: Left ventricle ejection time
MAP: Mean blood pressure
MRI: Magnetic resonance imaging
NPV: Negative predictive value
OSA: Obstructive sleep apnea
PAOP: Pulmonary artery occlusion pressure
PEP: Pre-ejection period
PPV: Positive predictive value
ROC: Receiver-operating characteristic
SBP: Systolic blood pressure
STR: Systolic time ratio
SV: Stroke volume
SVI: Stroke volume index
TFC: Thoracic fluid content
TFI: Thoracic fluid index
TN: True negatives
TP: True positives
VEPT: Volume of electrically participating tissue
VI: Aorta velocity index
VSR: Vascular systemic resistence
VSRI: Vascular systemic resistance index
